# Correlation between Crystal Structure, Surface/Interface Microstructure, and Electrical Properties of Nanocrystalline Niobium Thin Films

**DOI:** 10.3390/nano10071287

**Published:** 2020-06-30

**Authors:** L. R. Nivedita, Avery Haubert, Anil K. Battu, C. V. Ramana

**Affiliations:** 1 Center for Advanced Materials Research, University of Texas at El Paso, 500 West University Avenue, El Paso, TX 79968, USA; nivedita_lr@outlook.com (L.R.N.); akbattu@miners.utep.edu (A.K.B.); 2 Department of Physics, University of California, Santa Barbara, Broida Hall, Santa Barbara, CA 93106, USA; averyhaubert@ucsb.edu; 3 Department of Mechanical Engineering, University of Texas at El Paso, 500 West University Avenue, El Paso, TX 79968, USA

**Keywords:** niobium, thin films, nanostructure, microstructure, electrical properties

## Abstract

Niobium (Nb) thin films, which are potentially useful for integration into electronics and optoelectronics, were made by radio-frequency magnetron sputtering by varying the substrate temperature. The deposition temperature (*Ts*) effect was systematically studied using a wide range, 25–700 °C, using Si(100) substrates for Nb deposition. The direct correlation between deposition temperature (*Ts*) and electrical properties, surface/interface microstructure, crystal structure, and morphology of Nb films is reported. The Nb films deposited at higher temperature exhibit a higher degree of crystallinity and electrical conductivity. The Nb films’ crystallite size varied from 5 to 9 (±1) nm and tensile strain occurs in Nb films as *Ts* increases. The surface/interface morphology of the deposited Nb films indicate the grain growth and dense, vertical columnar structure at elevated *Ts*. The surface roughness derived from measurements taken using atomic force microscopy reveal that all the Nb films are characteristically smooth with an average roughness <2 nm. The lowest electrical resistivity obtained was 48 µΩ cm. The correlations found here between growth conditions electrical properties as well as crystal structure, surface/interface morphology, and microstructure, could provide useful information for optimum conditions to produce Nb thin films for utilization in electronics and optoelectronics.

## 1. Introduction

Today’s world is increasingly shaped by information technology and the need for advanced materials with enhanced properties and performance is ubiquitous in the current scenario [1,2,3,4,5,6,7]. The technical advancements in the recent years have paved way for miniaturization of electronic, magnetic, and optoelectronic devices with high performance, which is fundamentally dependent on the physical, chemical, and electromagnetic properties of their respective electronic, optical, magnetic, and electromagnetic components [1,2,3,4]. As such, the scientific research and development community challenged globally to investigate and improve the material properties for industrial or device applications that calls upon the persistent research and analysis of fundamental properties of materials [5,7]. This is important since it gives a clear understanding of the underlying mechanisms that governs the properties that are of direct relevance for the desired technological application. In most applications, electrically conducting or mechanically protective materials used are in the form of thin films, where improved efficiency and cost effectiveness is important [6].

Due to interesting electronic, chemical, and mechanical properties, refractory metals, especially Nb, Mo, and W, continue to dominate the aerospace, defense, electronics, and energy sectors [8,9,10,11]. Niobium (Nb) and Nb-based alloys exhibit quite interesting properties and are utilized in a wide variety of applications for industry. Niobium exhibits excellent physical and mechanical qualities which include density, young’s modulus, and yield strength of 8.6 g cm^−3^, 103 G Pa, and 240–550 N mm^−2^, respectively [11], making this material of notable interest for many technological and scientific applications where materials and devices often subjected to extreme environments. Similar to other refractory metals, Nb is also a metal with considerable hardness. In addition, Nb has been widely employed in microelectronics and optoelectronics owing to excellent electronic, thermal, and mechanical qualities. Thin films of Nb have become very appealing to the scientific community as a result of their numerous technological use such as nano-SQUIDs [12,13,14] circuits for switches operating at high frequency in cryogenic conditions [15], superconductivity [16,17], single photon detectors [18], and high temperature plasmonic applications [19,20,21]. Furthermore, recently, Nb based structural alloys have the focus of intense research in view of the possibility to design unique high-entropy alloys for application in energy and aerospace applications. Especially, Nb-based alloys in the form of bulk and coatings predicted to fulfil the requirements of next-generation alloys, which can find utilization for operation in extreme environments [22,23,24]. The present work was performed to investigate the fundamental structure-property relationship in nanocrystalline Nb films. The motivation is to gain understanding concerning the crystal structure, surface/interface microstructure and electrical properties as function of processing conditions in a comprehensive manner. The ultimate goal is to establish a structure-property correlation and optimum conditions that can serve as a roadmap to produce device quality nanocrystalline Nb films for application in electronics and optoelectronics.

Numerous investigations exist in the literature for Nb thin films made by variety of physical vapor deposition methods. Li et al. [25] explored the effect of different deposition pressures on the microstructure and nano mechanical properties. The increasing deposition pressure increases grain size, thickness, roughness, and hardness while porosity seen to decrease continuously. The impact of working pressure on the material properties of Nb thin films was evident. De Freitas et al. [26] observed that the superconducting properties degraded with *Ts* > 373 K. The interdiffusion at the Si/Nb interface was attributed as the main cause of the degradation and quality. Influence of baking and sputtering process on the residual gas composition in the vacuum chamber on the stress, electrical properties, and morphology of Nb thin films was examined by Wang et al. [27]. High-pressure magnetron sputtering was used to deposit Nb thin films by Banerjee et al. [28]. It was demonstrated that the Ar pressure affects the crystallite size and subsequent lattice expansion. The lattice expansion observed was in agreement with a linear elasticity model proposed recently. Okolo et al. have described the effect on the material properties and structure of Nb thin films depending on the substrate material and associated pre-treatment [29]. Hazra et al. [30] evaluated the correlation between the structure and superconductivity of sputtered Nb films. A deterioration in superconducting properties of Nb occurs with lattice expansion. Gontad et al. reviewed how substrate temperature influence pulsed laser deposited Nb thin films. It was found that the higher substrate temperature improves the Nb film quality, which results in the low resistivity of the films [31,32]. Thus, from the aforementioned brief review and analysis, it is clear that significant attention is being directed to Nb thin films in general, and their superconducting properties in particular. Though a lot of researchers have explored the superconducting properties of Nb, very few have focused on the possibility of using Nb as a thermally stable contact material. Our interest in Nb thin films for electronic device applications is also derived from the consideration of using it as a thermal contact for high-strength dielectric oxides in power electronics. Some of the materials that are currently used as contact material, like gold (Au), nano-architectures exhibit unsatisfactory thermal stability [33]. The response of Au nanorods to thermal heating evidence structural changes, although the timescales may vary, even to temperatures on the order of ~250 °C while such nanoscale features are fairly stable to higher temperatures of 700 ± 50 °C under the laser-induced heating experiments [33]. An alternative way to improve the thermal stability is to use refractory materials. Refractory metals exhibit high chemical stability and can withstand high temperatures as well. There are reports of using tungsten (W) and molybdenum (Mo) for plasmonic applications and many groups have been investigating their feasibility as a thermally stable contact material as well [34,35,36].

The performance of thin metal and alloy films in technological applications is dependent upon a myriad of features, like thickness, surface and grain morphology, crystallographic nature, structural imperfections, chemical composition, and residual stresses [37,38,39]. The electronic and mechanical properties of the metal and alloy thin films fabricated using the physical vapor deposition methods, especially the well-adopted and industry compatible sputtering, depends on their processing parameters [40,41,42,43]. The conditions during sputter-deposition processing, like substrate temperature, working pressure, target-substrate distance and sputter power, are most relevant in influencing the thin film quality, structure, and properties [37,38,39,40]. Therefore, tailoring these processing parameters and understanding the structure, morphology and electronic properties will augment our capability to alter the properties and performance as per the requirement of a given technological application. Optimizing the most favorable parameters for growth is a challenge during the process of deposition as they depend on all these conditions. To obtain good quality and conductive Nb thin films, a clear and robust comprehension about the electrical properties, microstructure, crystal structure, and morphology is necessary. Herein, we examine the deposition temperature effect on the phase stabilization, crystal structure, microstructure and morphology, and electrical properties of sputter deposited nanocrystalline Nb films. The results and analyses paves way in establishing a size-phase-microstructure-electrical property correlation in nanocrystalline Nb films.

## 2. Experimental Details

### 2.1. Thin Film Fabrication

Niobium thin films were deposited on Si(100) wafers by magnetron sputtering. A 2” Nb metal target (Plasmaterials Inc., Livermore, CA, USA) was used for deposition. Details of the deposition system employed in this work for Nb films were described previously elsewhere [39,40]. The deposition processing parameters employed for Nb film fabrication along with the characteristics of the Si(100) substrates are summarized in Table 1. While the Si(100) wafers procured (University Wafer Inc., Boston, MA, USA) were not subjected to for further characterization in this work, the previously measured and verified nature of the wafers and their characteristics assure the quality of Nb films produced. The details of the film deposition are as follows. The chamber was vacuumed to a base pressure of ~7.5 × 10^−5^ mTorr. High purity argon flow was maintained at a flow rate of 40 sccm throughout the deposition. MKS mass flow meters were used to control Ar gas flow. The substrate to target distance was 8 cm. The sputtering power was set a constant at 100 W while the working pressure of Ar was maintained at 5 mTorr for all Nb depositions. For each and every deposition, pre-sputtering of the Nb-target was carried out for 5 min to remove any native contamination. During pre-sputtering, a retractable shutter was introduced between the Nb-target and the Si-substrate. The deposition was made for a total time of 15 min; the shutter was retracted during this film growth period. The substrate holder was also continuously rotated using a DC motor (6 rpm) so as to maintain uniformity in the deposited Nb film. The only processing variable was the deposition temperature (*Ts*), which was systematically varied in the range of 25–700 °C at the steps of 100 °C. This is to ensure that the samples fabricated are only under variable deposition temperature and to study the influence of substrate temperature on the growth behavior, crystal structure, phase, morphology and microstructure, and electrical properties of resulting Nb films. Therefore, and since thin film quality and properties are fully dependent on deposition rate, the sputtering pressure, sputtering power applied to Nb-target and target to substrate distance were kept constant throughout the experimental study. As such, the effect of deposition rate and/or sputtering gas pressure is not within the scope of the present study while the optimum parameters were chosen based on extensive work performed either on Nb films and/or other metal films as reported previously elsewhere [40,43].

### 2.2. Characterization

#### 2.2.1. X-ray Diffraction (XRD)

The grazing incidence X-ray diffraction (GIXRD) studies were carried out on Bruker D8 Advance (Bruker, Billerica, MA, USA) using Cu-Kα radiation (*λ* = 1.5418 Å) at room temperature. We employed the standard procedures for structural characterization of metal and alloy thin films as reported elsewhere [39,40]. However, for clarity and continuity, the details of the XRD measurements made is as follows. The measurements made in detector scan mode with the glancing incidence angle set at 1°, step size 0.02°, and scan rate of 0.6°/min for all the measurements. The lattice parameter (*a*) was calculated using the relation:

(1) $$a_{o}=\frac{d_{hkl}}{\left(h^{2}+k^{2}+l^{2}\right)}$$

where the *d*_hkl_ is interplanar distance measured (from the XRD patterns). The in-plane average crystallite size (*D*) was calculated using the standard Scherrer’s equation [44]:

(2) $$D=\frac{0.9\lambda }{\beta \cos\theta }$$

where *λ*, *β*, and *θ*, are wavelength of the X-ray, full width half maximum, and angle of diffraction peak and *h*, *k,* and *l* are the lattice parameters.

#### 2.2.2. Scanning Electron Microscopy (SEM)

Surface imaging analysis of Nb thin film samples on Si wafers, in the secondary electron imaging mode, was performed using a high-performance and ultra-high resolution scanning electron microscope (Hitachi S-4800, Dallas, TX, USA). In order to avoid charging, the samples were sputter coated with gold before imaging. For the purpose of interface imaging and microstructure analysis, fractured samples were prepared from as-deposited Nb films on Si substrates. The samples were mounted in a vertical fashion so that the Nb–Si interface i.e., cross-sectional view, can be imaged easily. 

#### 2.2.3. Atomic Force Microscopy (AFM)

The surface topology and roughness analysis were carried out by Atomic force microscopy (Nanoscope iiia, Santa Barbara, CA, USA) using a standard commercial tip (Bruker DNP-S10) with a spring constant of 0.24 N/m by raster scanning across the film surface. We adopted the experimental procedures and approaches, which were quite successful to characterize the surfaces of metal/alloy thin films, as reported widely in the literature from our previous works [40,45]. The instrument was calibrated prior to any measurements being taken. All the AFM images were acquired in ambient air using constant force mode and digitized into 512 pixels’ × 512 pixels’. A variety of scans were carried out at random localities on the film surface to analyze the AFM images and the topographic image data were converted into ASCII data for analysis The average surface roughness (*R_a_*) and root mean square roughness (*R_q_*) were derived for Nb thin films using [46].

(3) $$R_{a}=\frac{1}{L}\overset{L}{\underset{0}{\int }}\left|Z\left(x\right)\right|dx$$

where *Z*(*x*) refers to the function that analyses the surface profile with regard to height (*Z*) and position (*x*). (*R_q_*) is the standard deviation of the variance from the mean plane of these sampling points. These parameters provide more quantitative information on the effect of processing parameters for nanocrystalline Nb thin films deposited under variable deposition temperature. 

#### 2.2.4. Electrical Properties

Electrical resistivity of the fabricated thin film was measured by four probe measurement technique with equally spaced pressure contacts. The measurements were carried out using the four-point Van der Pauw configuration. The *V*–*I* characteristics were measured using Lakeshore CRX-4K probe station (Lakeshore Cryotronics, Inc., Westerville, OH, USA) at room temperature. The samples were held in place through pressure contacts and the probes used for measurement were ZN50R tungsten needle probes. A Keithley 2400 source measurement unit (SMU) (Keithley Instruments, Cleveland, OH, USA) was the source used. Mobility and sheet resistance values were obtained using Hall Probe measurement system (Ecopia HMS 3000; Bridge Technology, AZ, USA). The electrical parameters reported in this work are as measured on the deposited Nb films. None of the measurements were calibrated with either small amounts of NbO_2_ detected and/or those arising from Si(100) substrate. However, the large difference observed in the measured values of Nb films compared to those quoted on the Si(100) substrates allowed us to consider the electrical characteristics and their variation with deposition temperature are pertained to Nb films deposited under variable processing conditions.

## 3. Results and Discussion

### 3.1. Crystal Structure, Phase, Growth Mechanism and Lattice Strain

#### 3.1.1. Crystal Structure and Phase Stabilization

**Figure 1:**
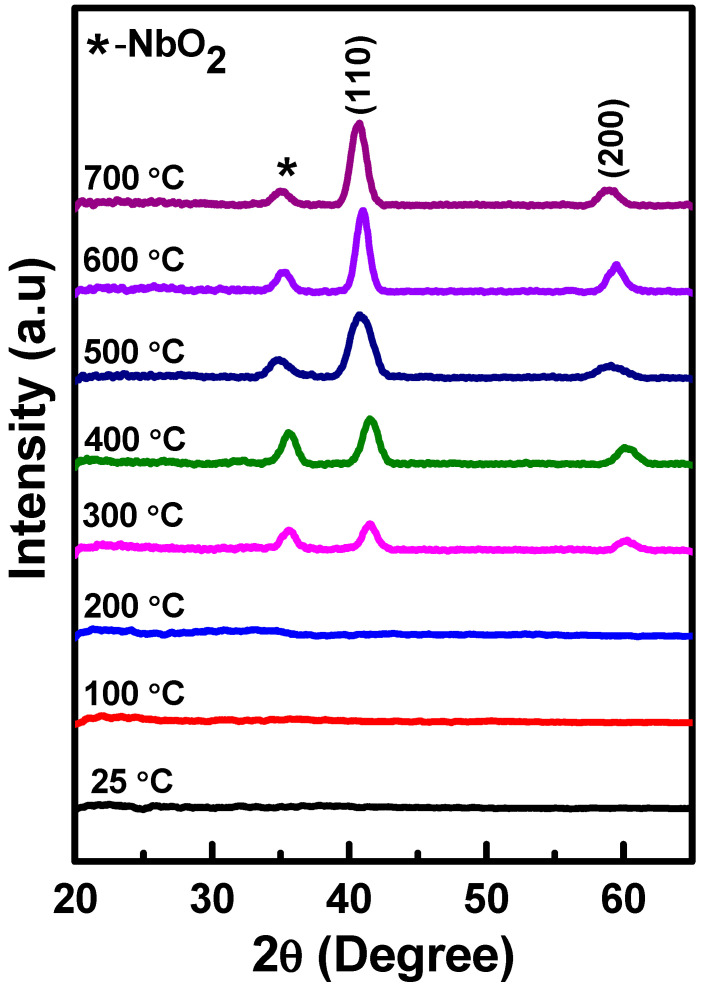
X-ray Diffraction (XRD) patterns of Nb thin films deposited at variable deposition temperature (25–700 °C). The effect of deposition temperature on the peak evolution is evident in the diffraction patterns. The smaller intensity peak at ~35° is due to surface oxide (NbO_2_) as indicated.

Figure 1 gives the XRD patterns for the Nb thin films deposited at various *Ts* (25–700 °C). The peaks observed at ~41° and ~58° corresponds to the diffraction from (110) and (200) planes specific to body centered cubic (*bcc*) Nb. The intensity of the (110) peak increases remarkably with increasing *Ts* which is an indication of improving crystallinity with temperature. It can be seen that, among the reflections noted, the (110) peak is dominant. Niobium crystallizes in *bcc* structure and, therefore, the (110) planes are the most densely packed while the surface energy is minimum for this plane with respect to other (*hkl*) planes. Perhaps, under the conditions of increasing thermal energy by means of deposition temperature, the growth of crystallites or grains along their (110) plane parallel to the film surface may be facilitated [29]. However, the presence of fairly broadened peaks in the XRD patterns could be due to the presence of crystallite size reduction i.e., smaller crystallites. The crystallite size calculated, using the Scherrer’s equation, varies in the range of 5 to 9 (±1) nm with increasing *Ts*. The additional peak, which is noted at ~35°, cannot be assigned due to *bcc* Nb but this peak matches and corresponds to (222) reflection of NbO_2_ [47]. The presence of NbO_2_ is, perhaps, due to surface oxidation that comes up as a resultant of high affinity of Nb towards oxygen and due to post deposition sample handling [25]. These naturally oxidized few monolayers were enough to passivate the surface and protect the bulk of the thin film from deeper oxidation. The samples were not stored in a desiccator and the fabricated thin films were left interacting with air atmosphere. 

The effect of deposition temperature on the growth behavior and crystallization of Nb films is evident from the XRD patterns shown in Figure 1. The Nb films deposited at *Ts* = 25–200 °C exhibit patterns without any indication of peaks. No presence of diffraction peaks in XRD patterns indicate that the Nb films deposited at 25–200 °C are amorphous in nature. The onset of XRD peaks, which were identified due to diffraction from (110) and (200) planes of *bcc* Nb, can be noted only for films deposited at 300 °C. The effect of *Ts* on the growth of Nb films may be explained as such. At lower temperatures, the interlude of atomic jump among the adatoms on the surface of the substrate can be large, resulting in the condensed species to be stuck at the deposited regions, ensuing amorphization of the deposited Nb films [43,47]. Increase in temperature facilitates the adatom mobility. [43,48]. At 300 °C, diffraction peaks emerges in the XRD pattern, indicating it as the critical temperature that promotes the nanocrystalline growth of Nb films, although the peaks are rather broad and the average crystallite size is ~5 nm. The deposition temperature is an important thermodynamic parameter that decides the crystal quality and phase stabilization in sputter-deposited films [39,41,42,43,48]. While the temperature required for realization of crystalline films depends on the specific material, for the current experimental conditions, a deposition temperature of 300 °C is provides sufficient energy, favorable for Nb crystallization at the nanoscale dimensions. 

#### 3.1.2. Lattice Parameter

**Figure 2:**
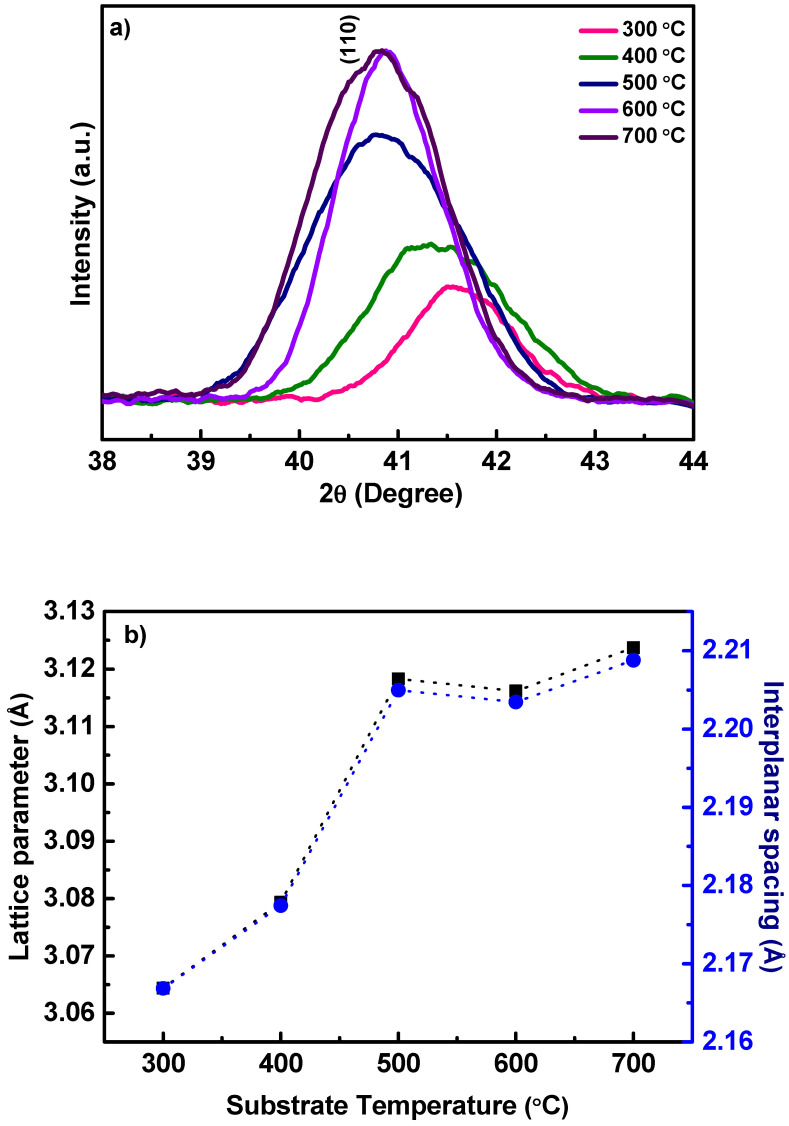
(**a**) High-resolution, detailed scans of (110) peak for Nb thin films deposited at varying substrate temperature. (**b**) Lattice parameter and interplanar spacing of Nb films deposited at varying deposition temperature.

Having understood the effect of *Ts* on the growth behavior and crystallization of Nb films, we then turned our attention to the details of lattice parameter and lattice strain (if, any). For this purpose, slow and detailed scans of (110) peak region of the Nb films was performed. The high-resolution, detailed scans of Nb(110) peak are shown in Figure 2a. It is evident that the (110) peak experiences a gradual, negative shift in the 2*θ* position with increasing *Ts*. Additionally, it can be seen that the peak becomes narrower with increasing *Ts*. The lowering of 2*θ* position is significant with increasing deposition temperature (Figure 2a). This observation indicates the stress in the Nb films with increasing *Ts*. The XRD results, thus, confirm the dependence of crystallinity and lattice parameter change with increasing *Ts*. To further confirm and to quantify the lattice expansion as a function of *Ts*, the lattice parameter of the Nb films was calculated based on the interplanar distance of (110) planes. The variation of lattice parameter of Nb thin films with *Ts* is shown in Figure 2b. It is evident that the lattice parameter of Nb film vary from 3.06 to 3.12 Å (lattice parameter of bulk Nb is 3.30 Å) as the growth temperature varies from 25 to 700 °C. 

It must be noted that the calculated lattice parameter, which is in the range of 3.06–3.12 Å, for nanocrystalline Nb films is relatively lower than the bulk Nb crystal (3.30 Å). The lattice parameter (*a*) variation with crystallite size (*D*) reduction has been widely reported in the literature [28,49,50,51,52,53,54]. However, there are evident cases of either lattice parameter increase or decrease as a function of the crystallite size and also depending on specific set of materials [28,49,50,51,52,53,54]. Several theoretical efforts and models exists in the literature, which describe the *a*–*D* correlation in nanocrystalline materials, especially metals and alloys [49,50,55,56,57]. The lattice parameter increases or decrease in nanocrystalline materials depends on several factors, such as: (a) the surface energy [55], (b) the lack of the outermost bonding of the surface atoms [55,56], and (c) intra-crystalline pressure [57,58]. Therefore, the relatively lower values of nanocrystalline Nb films compared to bulk crystal may be due to the effect of nanoscale dimensions. Ag nanoparticles with the size range of 3–18 nm exhibited reduction of lattice parameter with decreasing particle size [59]. On the other hand, Ag prepared using magnetron sputtering [60] demonstrates the extreme dependence of the lattice parameter, namely, the *a*-increase with decreasing *D* value to 12 nm. The nearest neighbor distance reduction and unit cell contraction with average crystallite size has been observed for nanocrystalline particles of several metals, such as Au, Ag, Ni, Pd, and Cu [49,52,59,60]. Thus, in the present case of Nb films, the lattice parameter increase with *Ts* is an indication of lattice expansion with respect to deposition or growth temperature. The difference in lattice parameter accounts for the lattice distortions in the crystal structure which inadvertently gives rise to residual strain in the thin films, which in this case is tensile in nature.

#### 3.1.3. Texture

The (110) texturing of nanocrystalline Nb films is evident in XRD studies. Furthermore, the degree of orientation increases with increasing *Ts* as is evident in the intensity increase in (110) peak intensity. The observed (110) texturing of Nb films can be explained as follows. The preferred orientation or texturing is influenced by the adatom mobility in a growing thin film. The correlation between texture development and processing parameters are explained by various models, like thermodynamic or growth kinetic models in physical vapor deposition methods [61,62,63]. Orientation in metal thin films can be accounted to two types of surface diffusion. Firstly, surface diffusion amid planes which is prominent when surface adatom mobility is low. The preferred orientation is dictated by the plane, usually high-surface energy plane, with lower surface diffusivity analogous to the substrate, and the crystallites grow. Secondly, surface diffusion among grains at the initial stage of growth. This mode of growth is dominant when there is high surface adatom mobility, consequently, the texture is along low surface energy plane [61,62,63]. Therefore, (110) texturing of Nb films deposited at *Ts* = 400–700 °C, is because of minimization of energy as observed from XRD data. The (110) planes, with lowest surface energy, are the closest-packed in *bcc* Nb. Hence, in order to aid in the formation of crystalline films, as per the given conditions of pressure and deposition temperature, the growth of *bcc* Nb with crystallites in (110) is thermodynamically preferred. Sputter deposited W and Mo films have previously reported (110) texturing [39,43]. Furthermore, in polycrystalline or nanocrystalline materials (ceramics, alloys, and metals) there is anisotropy and for different crystallographic directions, the strain energy densities will differ. It was demonstated by our group that those orientations with lower strain energy density will favor crystal growth, in a variety of materials deposited by physical vapor deposition [39,43,48] Therefore, under optimum deposition pressure, growth along (110) plane is favored by the increasing *Ts* in Nb thin films, while minimizing the strain-energy.

#### 3.1.4. Lattice Strain and Stress

**Figure 3:**
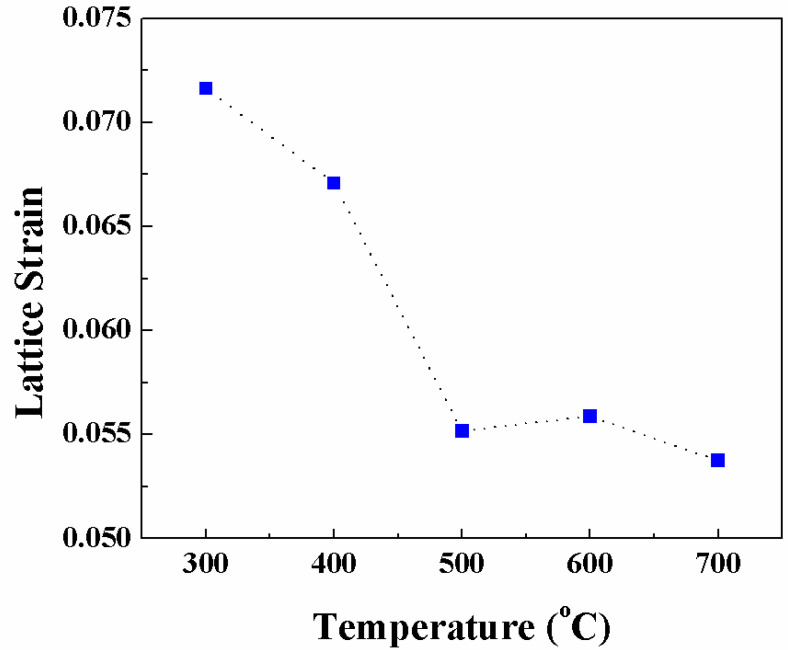
Strain variation with deposition temperature in Nb films.

During deformation the lattice spacing values are expected to change and the resulting elastic lattice strain ε^hkl^ is given by:

(4) $$\varepsilon =\frac{\Delta a}{a}$$

where ∆*a* = *a*_o_ − *a* and *a* is the change in lattice parameter and *a*_o_ corresponds to lattice parameter of Nb metal. Therefore, an increase in lattice spacing (tension) is indicated by positive lattice strain while negative strain is denoted by *a* decrease (compression). Lattice strain calculated from XRD data of Nb films deposited at various *Ts* is presented in Figure 3. It is evident that the lattice strain varies in the range of ~5–8% in the Nb films. Also, the trend noted reveals that the lattice strain decreases continuously with increasing *Ts*. Peak broadening in XRD patterns denotes small crystallite sizes or presence of lattice strain. Several factors like defects, incorporation of impurity atoms dislocations, micro- and macro stresses, promote to the strain induced peak broadening [8,11,34]. Thus, in nanocrystalline thin films, lattice strain intrinsically influence their properties. Figure 3 reveal the presence of strong internal stress in Nb thin films. It was revealed that the Nb films have small crystallites and usually in such crystallites, the dislocations are unstable and will congregate at the grain boundaries, which negates the possibility of dislocation contributing to strain. However, as grain size increases, the influence on dislocation stability by stress filed from the grain boundaries weakens, drastically increasing the likelihood of finding dislocations, thus, rendering their contribution to lattice strain plausibly considerable. Therefore, it may be deduced that interface tension can play a part in inducing stress in case of extremely small grains that results in excess volume of grain boundaries, while effect of dislocations becomes predominant in case of larger grains. Nevertheless, the lattice strain in nanocrystalline materials is predominantly due to the stress induced by the excess volume. 

### 3.2. Surface/Interface Microstructure

**Figure 4:**
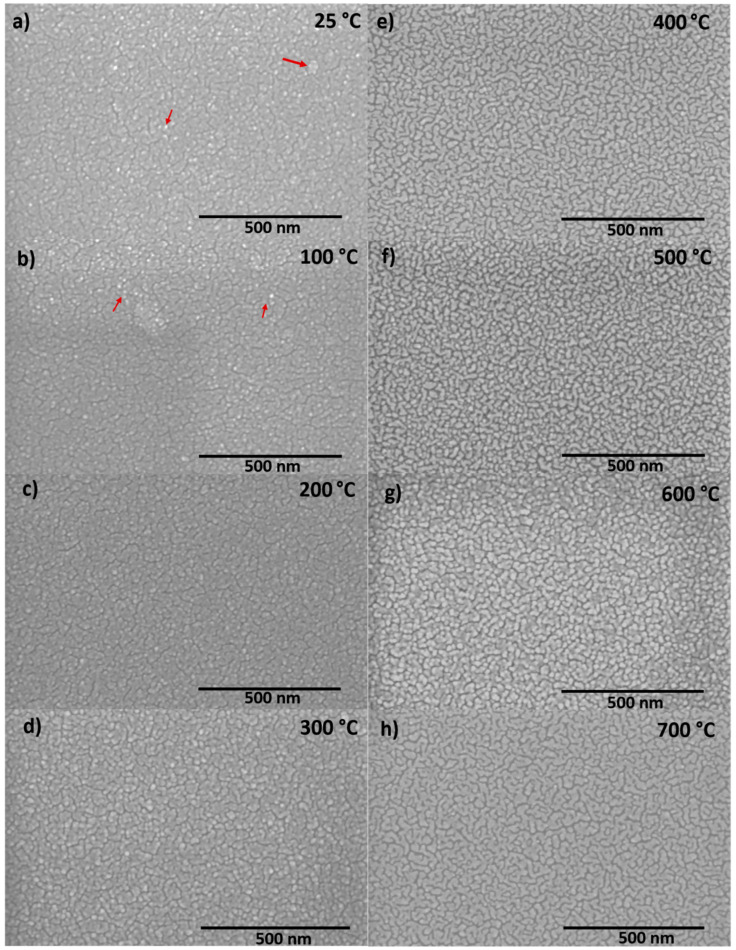
Top view scanning electron microscopy (SEM) micrographs of Nb thin films deposited at varying substrate temperatures of 25–700 °C. The data shown are for Nd films deposited at: (**a**) 25 °C, (**b**) 100 °C, (**c**) 200 °C, (**d**) 300 °C, (**e**) 400 °C, (**f**) 500 °C, (**g**) 600 °C and (**h**) 700 °C.

**Figure 5:**
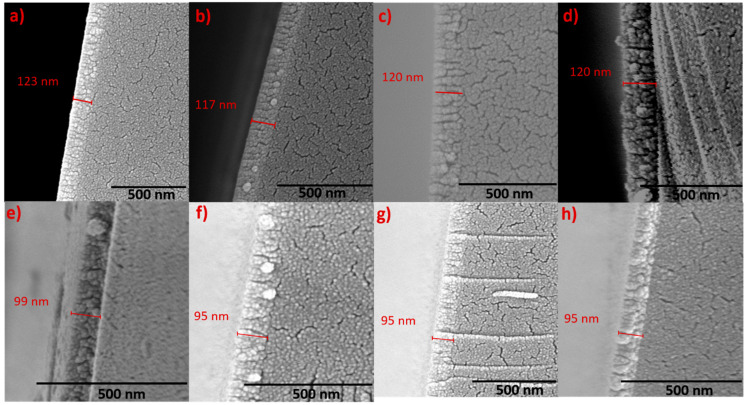
Fractural cross-section SEM micrographs of Nb thin films deposited at varying substrate temperatures of 25–700 °C. The data shown are for Nd films deposited at: (**a**) 25 °C, (**b**) 100 °C, (**c**) 200 °C, (**d**) 300 °C, (**e**) 400 °C, (**f**) 500 °C, (**g**) 600 °C and (**h**) 700 °C.

Figure 4a–h shows the surficial images of the Nb films at various *Ts*. The surface morphology of these Nb films appear to be uniform without any irregularities. All the Nb films exhibit similar grain structure, which could be described of possessing interlinking wave like a structure, which becomes more defined with increasing deposition temperature. At lower *Ts*, the presence of particles (indicated by the red arrows in Figure 4) that has not completely evolved into the wavy-grain can be seen in Figure 4a–c. As the temperature increases, the additional thermal energy provided to the migrating species will facilitate the growth and will result in more and more well-defined grains with dense arrangement. The cross-sectional SEM images indicating the interface microstructure of the Nb films are shown in Figure 5. The film(Nb)–substrate(Si) interface can be distinctly observed from the micrographs. Furthermore, the microstructure of Nb films is characterized by a columnar structure as is evident from crosssectional SEM images. Columnar growth is commonly observed in metal thin films deposited by sputtering. Specifically, the growth of the metal films will be a combination of nucleation as well as shadowing effect. Initially as the impinging atoms reaches the substrate surface they form isolated islands. These initial nucleated islands will behave as shadowing centers now, routing more impinging atoms to the taller islands as compared to shorter ones. This results in the taller islands to grow more in length leading to columns and thus columnar grain growth. While deeper investigation is not made by using more sophisticated analytical tools such as transmission electron microscopy or high-energy electron diffraction, as evident from XRD studies, the columnar grains of Nb films orient in such way so that the Nb film surface exhibit a strong (110) texturing. Also, the dense morphology leading to visible changes in columnar grain structure as function of increasing deposition temperature is evident.

**Figure 6:**
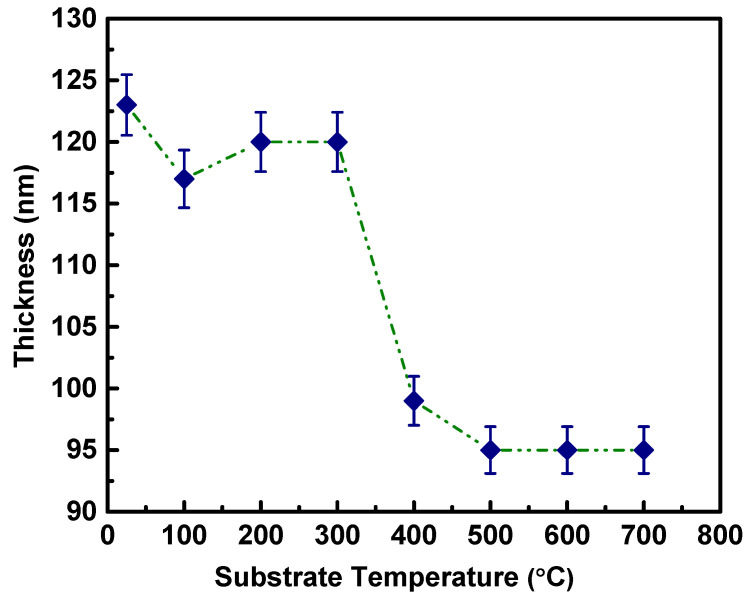
Variation of Nb film thickness with deposition temperature (25–700 °C).

The thickness of Nb decreases as the deposition temperature increases. The thickness of the samples are in the range of 100–120 (±5) nm. For Nb films deposited at room temperature, the thickness was found to be ~124 nm, which then continuously decreases with increasing *Ts*. However, while there was not much of a decrease in thickness for Nb films deposited at other intermediate *Ts*, film thickness reduces to ~99 nm at 400 °C. Finally, Nb film thickness decreases to ~95 nm with a further increase in *Ts* to 500–700 °C range. The functional dependence of Nb film thickness on *Ts* is presented in Figure 6. The decrease in thickness can be explained due to the increased mobility of the sputtered species with respect to thermal energy, leading to densification and compaction. The surface morphology of the higher temperature deposited thin films also exhibited well defined grains, with uniform, smooth morphology which conform to the densification of Nb thin films with respect to growth temperature.

### 3.3. Surface Topography and Morphology

**Figure 7:**
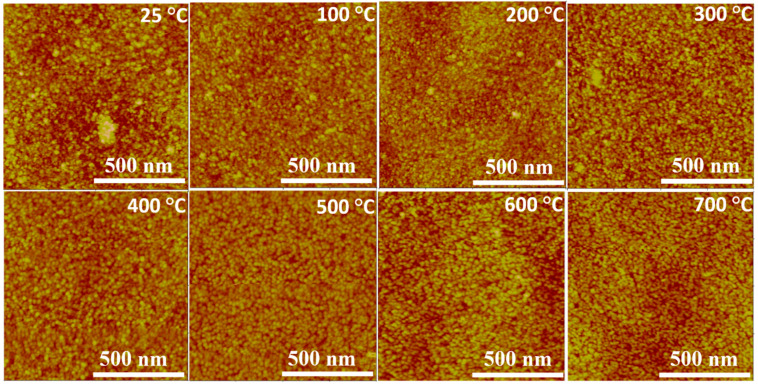
Topographic Atomic Force Microscopy (AFM) micrographs of Nb films deposited at variable *Ts*.

Figure 7 shows subtle but noticeable variations in the crystalline structures produced at different temperatures. Niobium samples deposited at *Ts* ≤ 300 °C have noticeable large crystalline grains, while at *Ts* > 300 °C there is a decrease in the enlarged crystalline structures and they are replaced by smaller elongated areas with increasing voids. This correlates well with SEM images (Figure 4) which show increasing boundary edges produced at *Ts* = 400–600 °C, while at 700 °C there are more elongated grains with smoother surface but still well-defined boundary edges. 

**Figure 8:**
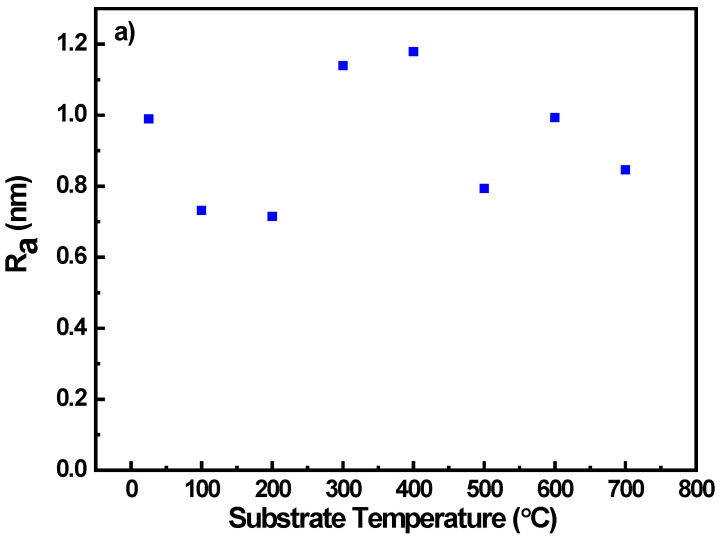

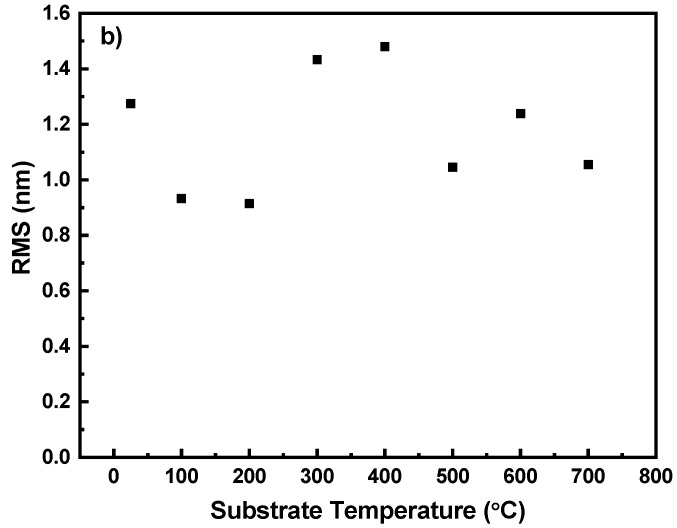
Variation of AFM surface roughness characteristic parameters of Nb films with *Ts*. Data shown are variation in: (**a**) average roughness (*Ra*) and (**b**) the Root Mean Square (RMS) roughness values with *Ts*.

The single numerical parameters, surface roughness (*Ra*) and root mean square (RMS), are useful when classifying surfaces of the same type of material that are produced under the same procedure [46]. Ra can be defined as the mean value of the surface height relative to the center plane and RMS defined as the standard deviation of the surface height within the given area [46]. The average roughness (*Ra*) and the Root Mean Square (RMS) are correlated well in this set of Nb films as seen from Figure 8a,b. Analysis from the surface topography shows that the Nb films produced were comparatively smooth with a root mean square (RMS) roughness value of 1.5 nm, where previously RMS values of 1 nm have been shown [64]. Higher *Ts* results in increase in thin film thermal energy, thereby enabling higher ad-atom mobility, providing smoother thin films.

**Figure 9:**
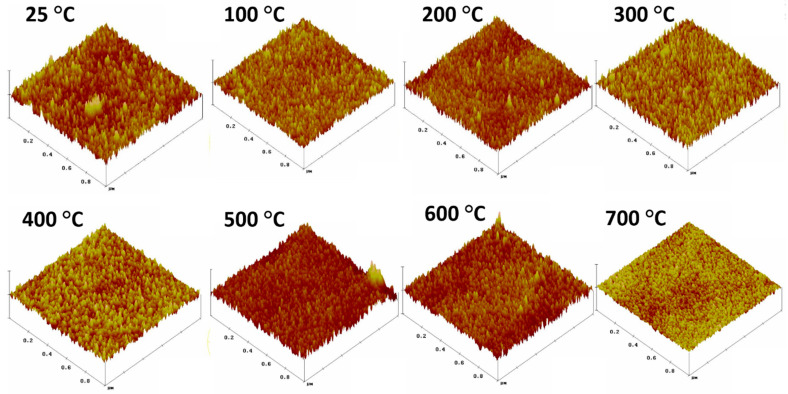
Three dimensional AFM micrographs of Nb films deposited at variable *Ts*.

**Figure 10:**
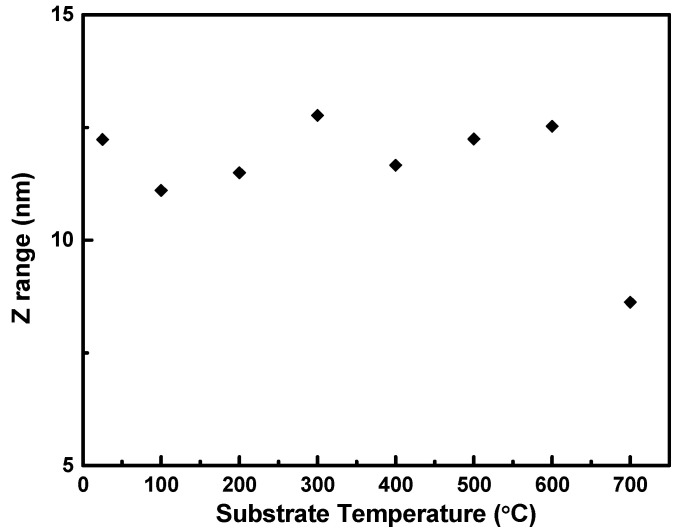
Measured height from roughness micrographs of Nb films at different temperatures (25–700 °C).

In Figure 9, the surface roughness profile for the thin film fabricated at different temperatures illustrates the z range to have high correlation with the RMS and Ra shown in Figure 8. The height profile of the films produced at different temperatures, given in Figure 10, are relatively similar and shows no linear correlation to deposition temperature as seen from Figure 9, and it can be observed that the smoothest film was produced at 700 °C. Consequently, we show here that the film height profile is lowest at 700 °C. 

### 3.4. Electrical Properties

The resistivity of any metal thin films depends on a myriad of factors like grain size, deposition temperature, surface roughness, etc. The resistivity of Nb thin films fabricated at different substrate temperatures was calculated from the current–voltage (IV) data obtained by four probe measurements and the following equation was used to calculate the resistivity.

(5) $$\rho =4.532\times R_{s}\times t$$

where *R_s_* is the sheet resistance and *t* is the thickness of the thin film. 

**Figure 11:**
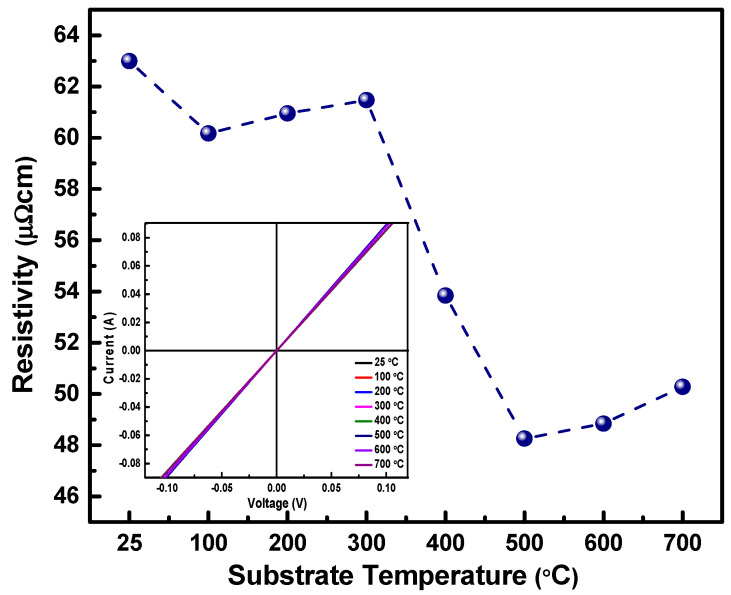
Resistivity dependence of Nb thin films on the deposition temperature. Inset shows the IV characteristics of Nb films.

The current–voltage (IV) characteristics of Nb films are shown in Figure 11. The current increases linearly with increasing voltage applied as seen in the inset of Figure 11. This linear trend in nature demonstrates perfect ohmic behavior, which is characteristic of metals. Figure 11 represents the variation in resistivity of Nb thin films with respect to substrate temperature Films deposited at higher substrate temperature exhibit lower resistivity as compared to those deposited at lower temperatures. The thin films deposited at 500 °C have the lowest resistivity of 48 µΩ cm. The overall resistivity values obtained from the Nb thin films are in the range of 48 to 63 µΩ cm. These values are comparative to the reported bulk values of Nb metal, which is 15 µΩ cm [65]. It is also interesting to note that the resistivity depends on thickness as well. The thin films with lower thickness register lower resistivity. The thickness of the films deposited at 500–700 °C is ~95 nm. It can be observed that there is a slight increase in the value of resistivity with increase in deposition temperature from 48 to 50 µΩ cm. This variation in electrical properties could be correlated to the microstructural properties of these films. The electrical properties of the thin film depend on the way the electrons interact with the structural imperfections of the thin film like the grain boundaries, micro strain, and other defects that could lead to electron scattering that determines the electrical properties like resistivity in metallic thin films [66]. In thin films, as the growth temperature increases, it facilitates the grain growth. The bigger the grains, the lesser the grain boundaries. When the grain boundaries, which act as a hindrance to the electron propagation in the thin film, is less as observed in higher temperature deposited thin films, the resistivity lowers, since it increases the mean free path for the electrons. 

The carrier density and degree of disorder of the thin films influence their transport properties. Standard Van der Pauw configuration is used for transport property measurement. Through hall measurement, the carrier concentration, type of charge carriers, mobility, sheet resistance and hall coefficient could be calculated. In the Van der Pauw configuration, current is applied to the sample through four pressure contacts, placed equidistant from each other, generally at the periphery of the sample. A uniform magnetic field of 0.57 T is applied to the samples during the measurement. A current of known value is traversed through the sample and the voltage across the two electrodes is measured while alternatively reversing the polarity. The resistivity and other related parameters are derived from a combination of eight measurements which are given by the following equations:

(6) $$\rho_{A}=\frac{\pi }{ln2}f_{A}t_{s}\frac{\left(V1-V2+V3-V4\right)}{4I}$$

(7) $$\rho_{B}=\frac{\pi }{ln2}f_{B}t_{s}\frac{\left(V5-V6+V7-V8\right)}{4I}$$

(8) $$\rho_{Avg}=\frac{\rho_{A}+\rho_{B}}{2}$$

where, $\rho_{A}$ and $\rho_{B}$ are resistivity in Ω cm, $t_{s}$ is the thickness in µm, V1–V8 are the measured voltages, *I* is the current through the sample in *A*, $f_{A}$, and $f_{B}$ are geometrical factors based on sample geometry.

The resistivity is given by:

(9) $$\rho =\frac{1}{en\mu }$$

where *n* is the free carrier concentration and *µ* is the mobility; which is given by the equation,

(10) $$\mu =\Delta R\times \frac{10^{8}}{B\;R_{sh}}$$

In the above equation, Δ*R* is the change in resistance resulting from the magnetic field (*B*) applied for measuring Hall voltages and $R_{sh}$ is the sheet resistance of the material [67]. 

**Figure 12:**
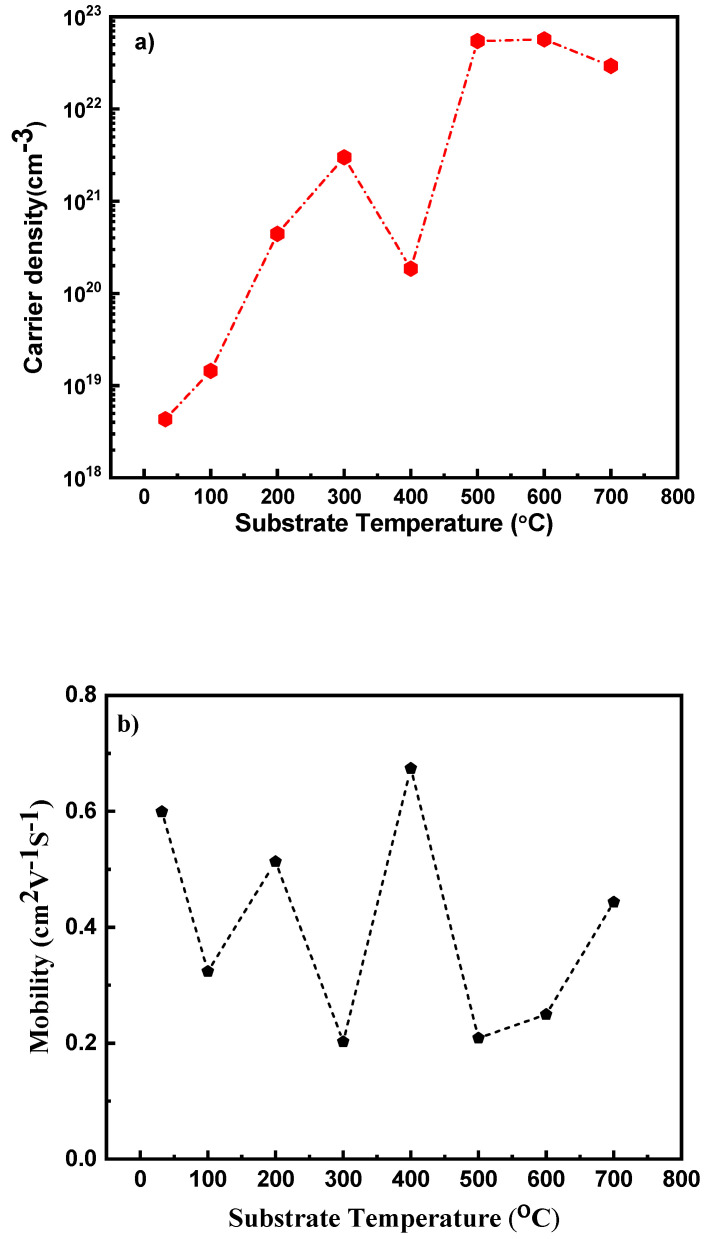
(**a**) Carrier density and (**b**) mobility of Nb films deposited at varying substrate temperatures.

Figure 12a,b gives the carrier density concentrations and mobility obtained from hall measurement, respectively. It can be observed that the carrier concentration increases at higher deposition temperatures. The resistivity obtained from the hall measurement value decreases with increase in *Ts*, which is in corroboration with the trend as observed from four probe measurement. The resistivity values obtained were in the range of 438–546 µΩ cm. However, the quantitative resistivity value shows multitudes of increase in the resistivity values at lower temperatures as compared to four probe measured counterpart values. Though there is a discrepancy at lower temperature, the resistivity values are comparable at higher substrate temperatures. 

It is important to specify the importance of studying the impact of processing parameters (in this case *Ts*) on the properties of Nb thin films. Nb is a technologically important material since it is widely investigated and used for its superconducting properties and potential high temperature applications. The results manifest that *Ts* strongly influences the structural, microstructural and electrical properties of the sputter deposited Nb thin films. The effect of *Ts* on the crystal structure and morphology of the Nb thin films could be ascribed to the thermally enhanced adatom mobility of the sputtered species. In the present work, we have noted that the *Ts* also play a significant role in tuning the electrical properties. The value of resistivity obtained in the current study is in the range of 48 to 63 µΩ cm, with carrier concentration as high as 5.705 × 10^22^/cm^3^. The low resistive Nb thin films obtained paves way to the prospective use of these thin films as an ohmic contact for electronic device applications.

## 4. Conclusions

Nanocrystalline Nb films were deposited by radio-frequency magnetron sputtering onto Si substrates. The extensive studies performed indicate that the effect of deposition temperature is significant on the surface/interface quality, morphology and microstructure, and electrical properties of Nb thin films. Niobium films deposited had amorphous nature at lower temperatures and nanocrystalline nature at *Ts* ≥ 300 °C, and stabilized in bcc phase of Nb. The crystallinity of the Nb film improves with increasing *Ts*. It was also observed that increase in substrate temperature also delivered thin films with increasing grain boundary edges with smoother surface. The growth temperature also dictated the resistivity of the fabricated thin films. The lowest resistivity observed was 48 µΩ cm, with a carrier concentration of 5.47 × 10^22^/cm^3^ and hall mobility of 0.2 cm^2^/Vs. The lower resistivity obtained in these thin films makes them feasible for use as a thermally stable contact material for use in electronics and power device applications.

## Figures and Tables

**Table 1:** Sputter-deposition processing parameters employed for Nb films.

Physical Parameter	Set Value
Base pressure Sputtering power	7.5 × 10^−5^ mTorr 100 W
Processing gases	Argon (Ar)
Substrates & Details	Silicon (Si) Si single crystal substrates, *n*-type; Resistivity:1~10 Ω cm Orientation (100) ± 0.5 deg Size: dia 50.8 mm (+/−0.1 mm) Thickness: 0.5 mm (+/−0.05 mm) Surface Roughness: <0.5 nm
Deposition temperature (*Ts*) Variability in *Ts* (Δ*Ts*) Target-to-substrate distance	25–700 °C 100 °C 8 cm
Total gas flow	40 sccm (constant)
Working pressure Pumping speed Deposition time	5 mTorr 50 L/s 15 min
Film thickness	~100 nm

**Remarks**: Substrates were rotated at speed of 6 rpm to ensure uniformity on the Nb films Si(100) substrates, which were cut from the large area substrates for the purpose of various measurements.
